# Differential Brain Expression Patterns of microRNAs Related to Olfactory Performance in Honey Bees (*Apis mellifera*)

**DOI:** 10.3390/genes14051000

**Published:** 2023-04-28

**Authors:** Jingnan Huang, Tianbao Wang, Yuanmei Qiu, Aqai Kalan Hassanyar, Zhaonan Zhang, Qiaoling Sun, Xiaomin Ni, Kejun Yu, Yongkang Guo, Changsheng Yang, Yang Lü, Hongyi Nie, Yan Lin, Zhiguo Li, Songkun Su

**Affiliations:** 1 College of Animal Sciences (College of Bee Science), Fujian Agriculture and Forestry University, Fuzhou 350002, China; 2 College of Life Sciences, Fujian Agriculture and Forestry University, Fuzhou 350002, China; 3 Laboratory of Evolution and Diversity Biology, UMR5174, University Toulouse III Paul Sabatier, CNRS, 31062 Toulouse, France; 4 Biological Science Research Center, Southwest University, Chongqing 400715, China; 5 Faculty of Science, University of Queensland, Brisbane, QLD 4072, Australia; 6 Mudanjiang Branch of Heilongjiang Academy of Agricultural Sciences, Mudanjiang 157041, China; 7 Academy of Bee Science, Fujian Agriculture and Forestry University, Fuzhou 350002, China

**Keywords:** *Apis mellifera*, olfactory performance, miRNAs, proboscis extension response (PER), brain

## Abstract

MicroRNAs (miRNAs) play a vital role in the nerve regulation of honey bees (*Apis mellifera*). This study aims to investigate the differences in expression of miRNAs in a honey bee’s brain for olfactory learning tasks and to explore their potential role in a honey bee’s olfactory learning and memory. In this study, 12 day old honey bees with strong and weak olfactory performances were utilized to investigate the influence of miRNAs on olfactory learning behavior. The honey bee brains were dissected, and a small RNA-seq technique was used for high-throughput sequencing. The data analysis of the miRNA sequences revealed that 14 differentially expressed miRNAs (DEmiRNAs) between the two groups, strong (S) and weak (W), for olfactory performance in honey bees were identified, which included seven up-regulated and seven down-regulated. The qPCR verification results of the 14 miRNAs showed that four miRNAs (miR-184-3p, miR-276-3p, miR-87-3p, and miR-124-3p) were significantly associated with olfactory learning and memory. The target genes of these DEmiRNAs were subjected to the GO database annotation and KEGG pathway enrichment analyses. The functional annotation and pathway analysis showed that the neuroactive ligand-receptor interaction pathway, oxidative phosphorylation, biosynthesis of amino acids, pentose phosphate pathway, carbon metabolism, and terpenoid backbone biosynthesis may be a great important pathway related to olfactory learning and memory in honey bees. Our findings together further explained the relationship between olfactory performance and the brain function of honey bees at the molecular level and provides a basis for further study on miRNAs related to olfactory learning and memory in honey bees.

## 1. Introduction

Honey bees *Apis mellifera* (*A. mellifera*) are social insects whose brain has a volume of less than 1 mm^3^ and comprises only approximately one million neurons [1]. Honey bees are excellent model organisms for studying learning and memory [2,3,4], and because they can distinguish colors, odors, and size [5,6,7]. The survival and development of honey bee colonies depends on food with olfactory performance being necessary for efficient foraging and greatly contributes to pollination services. During foraging, honey bees remember the smell of flowers through associative olfactory learning and fly between their hives and food sites. Proboscis extension response (PER), which is a conditioned response to conditional and unconditional stimuli, is typically used to investigate the olfactory learning behavior of honey bees under controlled laboratory conditions. Honey bees establish a link between odor and reward after several cycles of training [8,9,10,11].

Honey bees may encounter various environmental factors during their foraging activities. To date, most studies have demonstrated that MicroRNAs (miRNAs) participated in the formation and maturation of neurons. The miRNAs are considered essential regulatory factors in vertebrate and invertebrate learning and memory processes [12,13,14,15]. Previous studies on honeybee miRNAs revealed their key roles in mediating memory formation [16,17]. Thus, it is crucial to investigate whether miRNA is relevant for learning and memory behavior in honey bees. MicroRNAs, non-coding RNAs that are 18–23 nt in length, regulate gene expression at the post-transcriptional level through base pairing with target sites in mRNAs and participate in growth and development [18,19]. The miRNAs exert their effects by inhibiting protein synthesis or degrading target mRNA. A study demonstrated that miR-124 is an important regulatory factor for neurogenesis in *Drosophila melanogaster* [20]. Furthermore, most studies on miRNAs have focused on insect memory. A previous study systematically studied 134 miRNAs in *Drosophila melanogaster* and identified their roles in odor learning and memory formation [21]. In experiments to discriminate between naive and conditioned olfactory memory in *Drosophila melanogaster*, miR-276a was required in mushroom neurons for memory formation and in ellipsoid neurons for naive responses to odors [22]. In social insects, miRNAs modulate traits related to reproduction and labor division, and some Hymenoptera-specific miRNAs may contribute to the evolution of traits important for the evolution [23]. The role of miRNAs in insect behavior has been confirmed [24,25,26]. Using RNA interference technology, it was discovered that miRNAs play a crucial role in olfactory learning and memory in *Drosophila melanogaster* [21]. In *Drosophila melanogaster,* miR-124 is not expressed during muscle development but is expressed in the central nervous system [20]. In honey bees, miRNAs play a vital role in maze-based visual pattern learning and memory [27]. In forager bees, miR-11-3p and miR-281-3p are responsible for honey processing [28].

Numerous studies have been performed on the molecular mechanisms underlying odor-learning in honey bees, and some have found that 259 genes are differentially expressed after PER training [29]. The miR-276 and miR-1000 are highly expressed and mainly enriched in the Kenyon cells of mushroom bodies in honey bee brains. Therefore, these miRNAs are speculated to participate in the neurological function of honey bees [30]. The miR-932 may regulate memory in honey bees, and its loss results in the impairment of long-term memory formation in the brain but not memory acquisition. Moreover, it has been shown to interact with actin, affecting honey bee memory [16]. Blocking the function of miR-12 and miR-124 impairs early memory phases but does not affect gustatory sensitivity, habituation, and sensitization [17]. Lateralization of olfactory learning in honey bees demonstrated approximately 6% of protein-coding gene expression and more than two-fold changes in expression levels between the two hemispheres, with the right brain hemisphere having a higher number of differentially expressed genes. In contrast, more miRNAs are expressed more strongly in the left hemisphere [31]. In honey bees, miRNAs can influence learning and neuronal plasticity. Specifically, in honey bees, miR-124 participates in the formation of early memory [17] and plays a crucial role in pattern learning [27].

All these studies have proven that miRNAs are important regulatory factors in honey bee behavior. However, the exact mechanism by which miRNAs affect the odor-learning capabilities of honey bees remains poorly understood. In order to examine the potential link between miRNA and the olfactory performances of honey bees, we conducted experiments with 12 day-old honey bees using odor-associative learning training to distinguish strong (S), and weak (W) olfactory performance. Next-generation high-throughput small RNA sequencing (sRNA-seq) technologies were analyzed, and we investigated the global miRNA-based olfactory learning in the brains of honey bees. Differentially expressed miRNAs (DEmiRNAs) in honey bee brains, and their target genes of *A. mellifera* were analyzed. We investigated the relationship between differential miRNA expression and the olfactory performance of honey bees. Several miRNAs were differentially expressed in S and W learning honey bees. We screened out bees with different olfactory performances to explore the potential miRNAs related to olfactory learning in honey bees, providing new evidence for understanding the olfactory learning capabilities in honey bee brains.

## 2. Materials and Methods

### 2.1. Bees Preparation

Honey bees (Fengqiang No.1 Western honey bees, *A. mellifera*) were collected from the experimental apiary at the College of Animal Sciences (College of Bee Science), Fujian Agriculture and Forestry University. Sealed brood combs were extracted from three healthy colonies from the same location, and emerging bees were marked and transferred to the same hive. The sealed brood combs were confined in a cage and placed in a constant-temperature incubator at +34 °C and 70% relative humidity. Emerging honey bees were collected at 9:00 a.m. every day and marked with paints of different colors on their thorax. The marked honey bees were then returned to their original hives. Once the marked honey bees reached the age of 12 days, they were captured using a soft tweezer and placed in an air-permeable glass bottle [32]. The bottle was placed in crushed ice to render the honey bees motionless. Individual bees were immediately harnessed with a unique copper tube. The honey bees could move their proboscis flexibly but could not rotate their heads randomly. Finally, the fixed honey bees were placed in a constant temperature and humidity incubator at +30 °C and 70% relative humidity for 1.5 h [33,34]. Then, the odor-associative learning experiment was carried out.

### 2.2. PER Test and Olfactory Performance Evaluation

The bees were trained to discriminate a rewarding odorant (positive conditioned odorant A or CS+) from a non-rewarding odorant (negative conditioned odorant B or CS−) by a differential conditioning protocol during six trials (three CS+ trials and three CS− trials) [35,36]. Each training bee was exposed to the two odorants according to the pseudorandom order (ABBAAB). Before the start of training, a 50% sucrose solution was used to select candidate bees for subsequent PER. The antenna of each harnessed honey bee was touched with a drop of 50% sugar water (wt/wt), and the honey bee that did not extending its proboscis to sugar water stimulation was discarded from the test [37]. A total of 309 sucrose responsive individuals were obtained and trained, and each honey bee underwent six training trials. The sucrose solution was provided as a reward to the PER honey bees when using 1-nonanol as odor A, and 1-hexanol as odor B was provided as the control odor without the sucrose solution reward. The odor-learning training of a single honey bee lasted 39 s. First, the harnessed bee was positioned in front of an olfactometer (kindly provided by M. Giurfa, University of Toulouse, France) for 10 s before the PER test and then placed in an airflow without any odor for 5 s. Subsequently, the odor was supplied for 4 s as a conditional stimulus followed by a 2 s sucrose solution (30%) presentation, with the odor and sugar being overlapped for 2 s (Figure S1). Finally, clean air was given in the absence of other stimulations for 20 s to complete the trial. The intertrial interval was 10 min [38]. The CS− trials followed the same sequence but no sucrose was delivered during the odorant presentation. The olfactory performance of the honey bees was assessed based on their PER to odor. In this study, honey bees were considered to have achieved conditional-stimulus and unconditional-stimulus associative learning only when they performed PER immediately and presented a strong ingestion action when their antenna came into contact with the odor compound.

The olfactory performance of the bees was evaluated based on the individual acquisition scores obtained from the training on odor-association learning [39,40]. During training, honey bees that responded to odor A obtained a value of 1, while in the absence of a response a value of 0 was attributed. In the CS− trails, those that did not display PER for odor B received a value of 0 while a value of −1 was attributed upon incorrect responding. The bees that responded to the CS− were not considered for the analyses (defined as the fail-learners). The bees not responding to the sucrose at the end of the tests were discarded. The total scores were calculated in the last two CS+ and two CS− trials. Therefore, the score could vary between 0 and 2, where higher positive acquisition scores indicate efficient learning performances [41]. Here, honey bees with a total acquisition score of 2 points were considered to have a strong olfactory performance, and those with a score of 0 were considered to have a weak olfactory performance. After training, the honey bees were frozen in liquid nitrogen immediately, and their heads were collected and stored in an ultra-low-temperature refrigerator at −80 °C for subsequent brain dissection.

### 2.3. Brain Dissections

The honey bee brains were dissected at low temperatures on dry ice, and the samples were placed in RNase-free Eppendorf tubes with nine brains per tube with three biological replicates being considered for each group. The brain sample of honey bees with strong olfactory performance are denoted as S1, S2, and S3 (*n* = 3), whereas those of honey bees with weak olfactory performance are denoted as W1, W2, and W3 (*n* = 3). Each group comprised nine pooled samples (*n* = 54), which were randomly selected from the trained bees. In miRNA validation, three pooled brain samples were prepared by the same method we have mentioned above.

### 2.4. RNA Isolation

The total RNA was extracted from 9 bee brins pooled per samples using TRIzol reagent (Invitrogen, Carlsbad, CA, USA) according to the manufacturer’s instructions. The RNA degradation and contamination were monitored on 1% agarose gels. The RNA purity was checked using the NanoPhotometer^®^ spectrophotometer (IMPLEN, Westlake Village, CA, USA). The RNA concentration was measured using Qubit^®^ RNA Assay Kit in Qubit^®^ 2.0 Flurometer (Life Technologies, IMPLEN, Westlake Village, CA, USA). The RNA integrity was assessed using the RNA Nano 6000 Assay Kit of the Agilent Bioanalyzer 2100 system (Agilent Technologies, IMPLEN, Westlake Village, CA, USA). To verify the candidate miRNAs through qPCR verification the total RNA was extracted from three bee brains pooled from each sample with three replicates from eight samples for each miRNA.

### 2.5. cDNA Library Preparation for Small RNA Sequencing

A total of 3 μg of total RNA per sample was used as input material for the cDNA library construction. The sequencing libraries were generated using the NEBNext^®^ Multiplex small RNA Library Prep Set for Illumina^®^ (Set 1) (New England Biolabs (NEB), Ipswich, MA, USA), following the manufacturer’s recommendations. The clustering of the index-coded samples was performed on a cBot Cluster Generation System using TruSeqSR Cluster Kit v3-cBot-HS (Illumia) according to the manufacturer’s instructions. After the cluster generation, small RNA library preparations were sequenced on an Illumina Hiseq 2500 platform and 150 bp single-end reads were generated. Each group represented a single small RNA library of six small RNA libraries. Novogene Biotech Co., Ltd. in Beijing performed the small RNA sequencing. The original data were uploaded to the NCBI SRA database (BioProject No. PRJNA724895).

### 2.6. Quality Control and Assessment of Sequencing Data from Brains of Worker Sample

Perl scripts were used for quality control and location of sequencing data, which were compiled independently by Beijing Novogene Biotech Co. Ltd., to obtain the unannotated tags of miRNAs. The sequences of the unannotated tags were compared with those of the reference genomes (assembly *Amel*_HAv3.1) of *A. mellifera* using Bowtie software [42,43]. The reads of the reference sequence were compared with sequences in the designated range of miRBase20.0, to acquire the potential sRNAs and secondary structures, as well as the sequences, length, and number of occurrences of the miRNAs in different samples. The sRNAs were identified based on the annotation sequences using Repeat Masker and Rfam software. The transcript per million (TPM) algorithms were used to normalize the read counts expression of the miRNAs in different sample distributions. The input data for the DEmiRNAs for the read count data were collected through the analysis of miRNA expression levels (Table 1). The DEmiRNAs were screened based on the criterion of *p*-value < 0.05, |log2 fold change (FC)| ≥1) in accordance with a study based on DESeq2 with a negative binomial distribution [44].

### 2.7. DEmiRNA and Target Gene Analyses

The standard miRNA target prediction methods miRanda and RNAhybrid were used to predict target genes, and common target genes between the two systems were selected [45,46]. The results were represented as 1, indicating that the software predicted the target gene, and 0, indicating that the software did not predict the target gene. Multiple statistical tests were performed to identify the target genes after DEmiRNA identification. The hypergeometric test/Fisher’s exact test with *p*-value < 0.05 was considered significantly enriched, and the false discovery rate (FDR) correction method, which Benjamini and Hochberg conducted to detect the most enrichment pathway terms. Gene Ontology (GO) enrichment and KEGG enrichment analyses were performed on the sets of target genes of the DEmiRNAs in accordance with the corresponding relationship between the miRNAs and the target gene candidates. The GO pathway enrichment analysis of differentially expressed genes was performed using the cluster Profiler R package Pheatmap in which the gene length bias was corrected. The GO terms with a corrected *p*-value < 0.05 were considered significantly enriched by differentially expressed genes, while KOBAS software was used to test the statistical enrichment of the predicted target genes in the KEGG pathways [47].

### 2.8. Stem-Loop RT-qPCR Verification of the Stem Rings of DEmiRNAs

The stem-loop RT-qPCR verification was performed for all 14 differentially expressed miRNAs. The specific stem-loop primers, upstream primers, and universal reserve primers were designed using miRNA Design software (Vazyme, Nanjing, China). Housekeeping genes (U6) was used as an appropriate internal control to normalize the data and to calculate the fold changes. The mQ primer R was chosen as the universal primer, and the primers were synthesized using Sangon Biotech Co. Ltd., Shanghai, China. (Table S2). The extracted RNA samples were reverse-transcribed using miRNA 1st Strand cDNA Synthesis Kit by stem-loop, (Vazyme, Nanjing, China), and the acquired cDNA was used as the RT-qPCR template. The reaction and primer concentrations ranged from 5 µL of the SYBR Green, 0.2 µL of the universal downstream primer at a concentration of 2 µmol/µL, 0.2 µL of the universal downstream primer at a concentration of 2 µmol/µL, 1 µL of cDNA template, and 3.6 µL of RNase-free H_2_O. The temperatures conditions were as follows: 95 °C for 5 min, 95 °C for 10 s, and 60 °C for 30 s for 40 cycles. The melting curve condition was 65 °C for 0.5 s and 95 °C for 0.5 s. The relative gene expression was calculated using the 2^−∆∆CT^ method [48].

### 2.9. Statistical Analyses

The olfactory performance score was evaluated by counting the individual PER responses to the odor stimuli A and B, S with W learning. The RT-qPCR data statistical analyses were carried out using SPSS software (IBM) and GraphPad Prism5.0 software. The data are presented as mean ± standard deviation. An independent-sample *t*-test was used for the statistical analysis with a *p*-value ≤ 0.05. The GO-Seq (Release2.12) was used for the GO enrichment analysis, and KOBAS (v2.0) was used for the KEGG enrichment analysis and was defined as statistically significant.

## 3. Results

### 3.1. Quality Control

**Figure 1:**
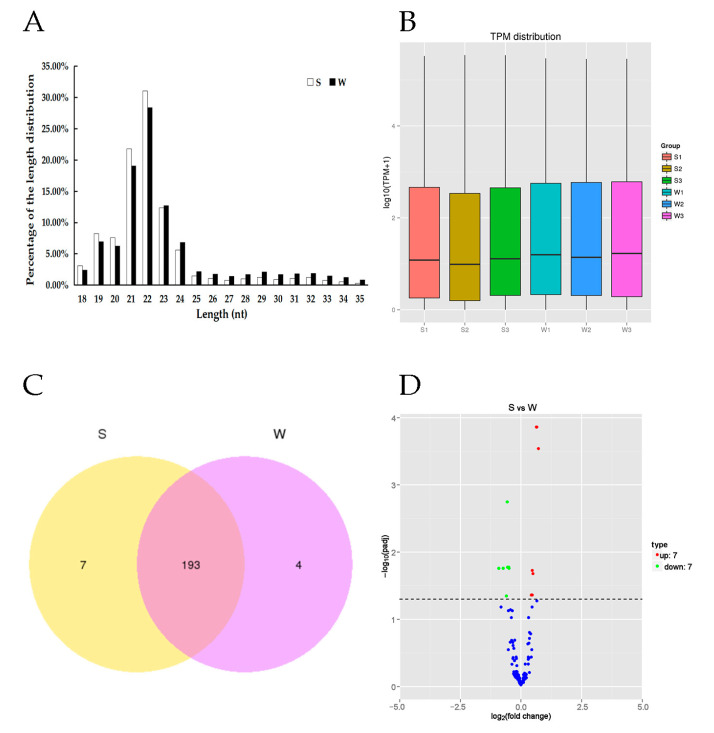
Distribution of length, transcript per million (TPM) distribution, Venn diagram and Volcano plot of miRNAs. (**A**) Distribution of the lengths of the miRNAs reads in the honey bee with strong or weak olfactory performance. The lengths of the miRNAs mainly ranged from 18 nt to 35 nt and peaked at 22 nt. (**B**) The y-axis represents log^10^TPM+1from three groups S, strong and W, weak performing learning and memory. (**C**) Venn diagram represent the total of 204 miRNAs identified in the two groups (S and W) of honey bees, of which seven unique in S and four unique in W and 193 common DEmiRNAs. (**D**) Differentially expressed miRNAs in the brain between strong olfactory performance and weak olfactory performance based on the fold change (FC) and the significance levels after correction.

A total of 64,063,581 raw reads were produced from the six cDNA libraries, and 62,400,830 clean reads were obtained after strict quality control (Table 1). The percentage of clean reads among the raw reads in each library ranged from 96.70% to 97.95%, with a mean Q30 of 91.50%. The miRNA lengths mainly ranged from 18 to 35 nt and peaked at 22 nt (Figure 1A,B). In this experiment, 12 day-old workers were chosen for odor-associative learning training, and three replication groups were set. A total of 309 effective individual bees were obtained. In total, 142 honey bees had a strong learning performance and 66 honey bees had a weak learning performance.

### 3.2. DEmiRNAs Analysis in the Honey Bee Brains

**Figure 2:**
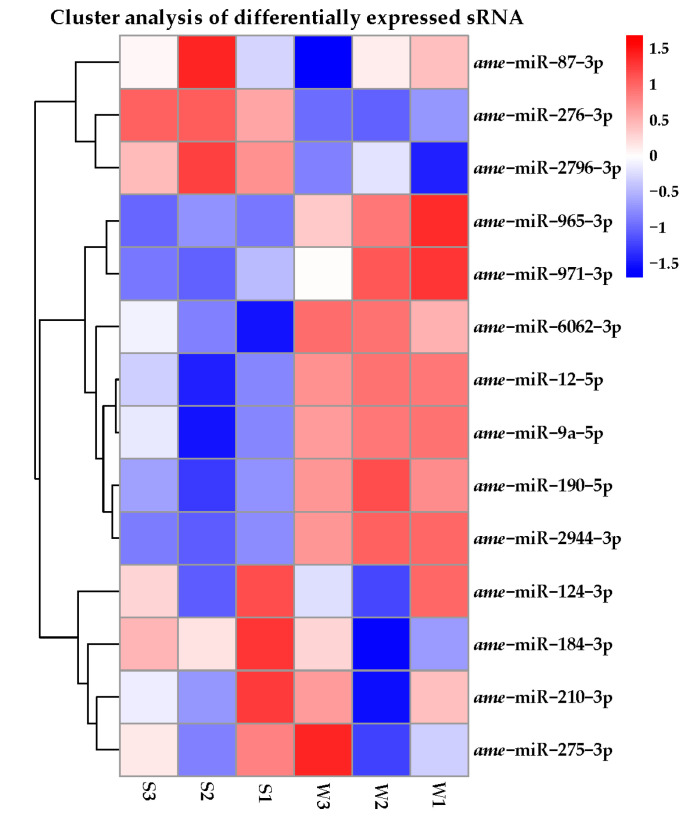
The overall hierarchical clustering diagram analysis of DEmiRNAs expressed. Differentially expressed miRNAs in the bee brains between strong olfactory performances was denoted as (S1, S2, and S3), and weak olfactory performance was denoted as (W1, W2, and W3), which is based on log10 transcript copies per million tags (TPM + 1) values, of the three groups of honey bee brains. Based on the fold change (FC) and the significance levels after correction. The blue color represents miRNAs with low expression, and the red represents miRNAs with high expression. The overall distribution of differential miRNAs were analyzed from two aspects: Fold change (FC) and corrected significance level. Levels were evaluated, and differential miRNAs were screened. When the sample has biological replicates, the differential miRNAs are filtered by PADJ < 0.05. When the sample is not biologically replicated, the number of differential miRNA will be large. In order to control the false positive rate, *q*-value is required to combine with FC selection, difference of miRNAs filter condition was the *q*-value < 0.01|log2 FC| > 1.

A total of 204 miRNAs were identified in the two groups of honey bees with different olfactory performance (Figure 1A). They included 176 known miRNAs, 28 novel miRNAs (Figure 1C) and 14 of which were differentially expressed miRNAs between S and W (Figure 1D). Among these DEmiRNAs, seven were up-regulated (miR-184-3p, miR-2796-3p, miR-210-3p, miR-87-3p, miR-124-3p, miR-275-3p, and miR-276-3p) in the S olfactory performance group, and seven were down-regulated (miR-2944-3p, miR-9a-5p, miR-6062-3p, miR-965-3p, miR-971-3p, miR-190-5p, and miR-12-5p) in the W olfactory performance group. These 14 DEmiRNAs were known miRNAs is shown (Table 2 and Figure 2).

### 3.3. DEmiRNAs and Target Gene Predicted Functional Annotation

**Figure 3:**
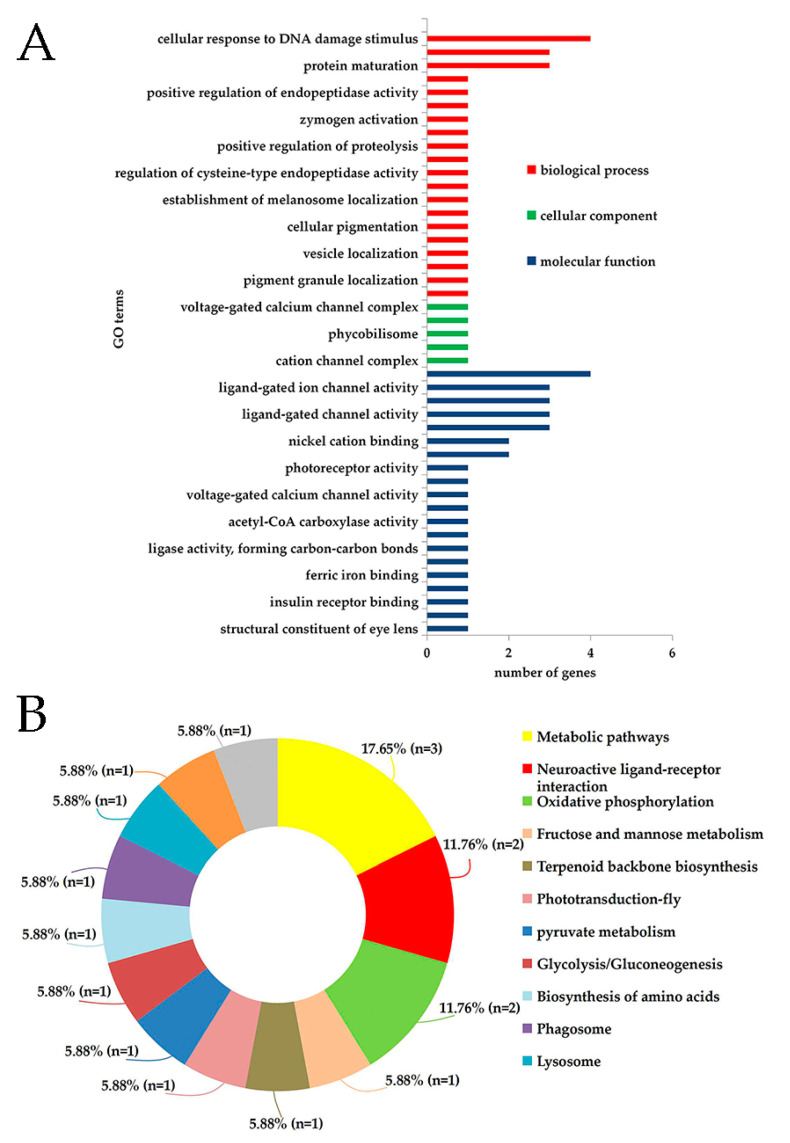
Gene Ontology (GO) enrichment analysis and Kyoto Encyclopedia of Genes and Genomes (KEGG) database of potential target genes of *A. melliferra* DE miRNAs annotation. (**A**) GO of potential target genes of the DE miRNAs of *Apis melliferra.* The DEmiRNAs were identified through the comparison of honey bees with strong olfactory performance with those with weak olfactory performance. The x-axis shows the GO term and the y-axis indicates the number of genes that were enriched in the three categories (biological process, cellular component, and molecular function). (**B**) KEGG database enrichment of potential target genes of the DE miRNAs of *Apis melliferra* were annotated to 13 pathways (Figure 3B). The scatter plot for KEGG enrichment results is shown in (Figure S2) and the mot enriched pathway terms is shown in (Table S1).

A total of 57 target genes were predicted for the 14 differentially expressed miRNAs (Table 2). The annotation results of the GO database showed that these 57 target genes were involved in 45 GO terms, including 20 biological processes (e.g., cellular response to DNA damage stimulus, protein processing, and protein maturation), five cellular components e.g., cation channel complex, ion channel complex, and 20 molecular functions e.g., gated channel activity, ligand-gated ion channel activity, and ligand-gated channel activity (Figure 3A).

### 3.4. RT-qPCR Verification of Bee Brain DEmiRNAs

**Figure 4:**
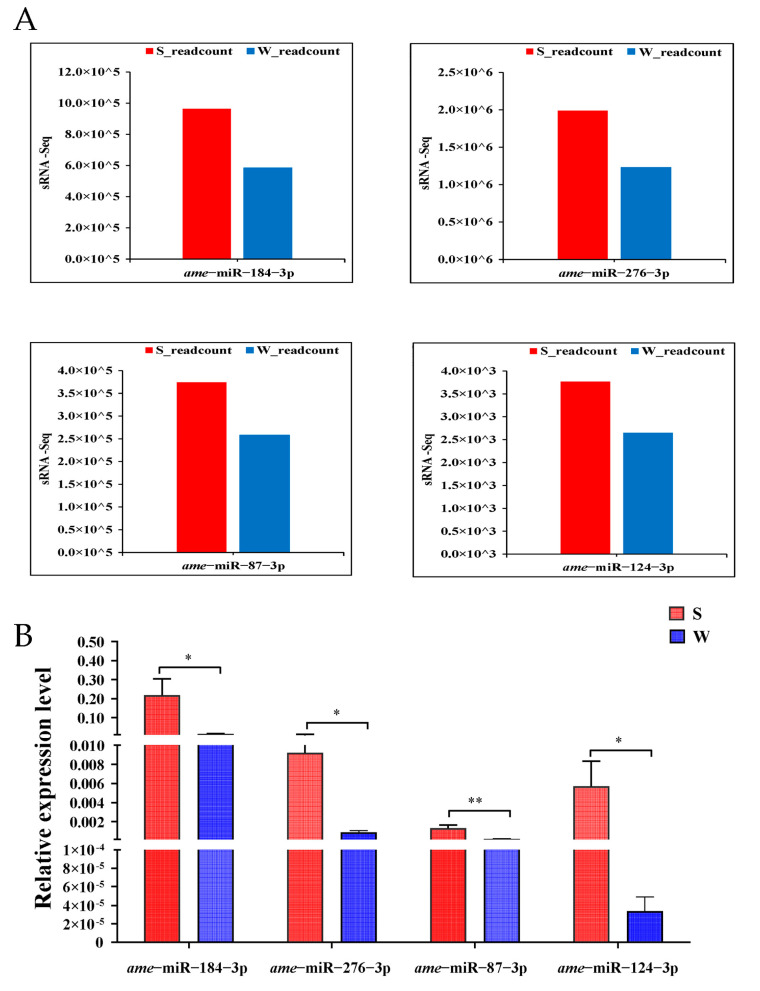
qPCR verification of four DEmiRNAs. (**A**) Shows four small RNA Seq and (**B**) shows the verified of the four miRNAs through qPCR. All 14 differentially expressed miRNAs were validated through qPCR the results showed that only four miRNAs (miR-184-3p, miR-276-3p, miR-87-3p, and miR-124-3p) were significantly associated with olfactory performance and memory. The relative expression data between the strong and weak olfactory performance groups (S/W) were calculated using 2^−∆∆CT^ method. The data were analyzed using the independent sample *t*-test with *p* ≤ 0.05, *n* = three pooled brains per sample and eight samples from each group. The stars represents the significant level e.g., *, *p* ≤ 0.05 and **, *p ≤* 0.005. The results of these four DEmiRNAs were consistent with small RNA-Seq.

The RT-qPCR further validated the relative expression levels of four DEmiRNAs is shown in (Figure 4). The results showed that the variation trend of the expression levels in the bee brains of these DEmiRNAs exhibited a similar trend between the qPCR results and the sRNA-seq data. In the S vs. W comparison, miR-87-3p, miR-276-3p, miR-124-3p, and miR-184-3p were up-regulated thus verifying the reliability of the sequencing results.

## 4. Discussion

In this experiment, the olfactory performance of honey bees was evaluated based on PER scores of odor association training. A high score was considered to be associated with a strong learning performance in honey bees and was used to screen honey bees with different capabilities [39,40,49]. In this study, we identified 14 differentially expressed miRNAs, which were significantly associated with olfactory performance and memory after PER and small RNA-seq technology, which showed that odor learning bees were strongly link to changes in different miRNA levels after conditioning. To verify the candidate miRNAs the specific primers (Table S2) were designed and 14 miRNAs were verified through qPCR. The results of the qPCR showed that 14 miRNAs were differentially expressed between S and W, one miRNA was not well amplified and was discarded from the statistical analysis, and nine miRNAs were showed significant differences between S and W (Table S3). However, based on the RNA-Seq only four miRNAs (miR-184-3p, miR-276-3p, miR-87-3p, and miR-124-3p) were significantly associated with olfactory learning and memory and these four miRNAs were consistent with the small RNA-Seq. Our findings provide information, new insight, and revealed and identified four DEmiRNAs which may participate in the olfactory performance and memory of honey bees.

In recent years, the role of miRNAs, and its participation in an insect’s neuromodulation has been well demonstrated [24,26]. Through a sucrose response experiment, it was found that in honey bees the overexpression of miR-279a may weaken responses to sucrose, and miR-279a plays a vital role in the division of labor [50]. Using sRNA-seq technology it was found that miRNA expression in the brains of honey bee individuals changed after visual learning and detected 40 known DEmiRNAs, including miR-124, miR-210, miR-971, and miR-9a. This result demonstrated that honey bees could induce a differential expression of different miRNAs after learning performance, and that specific miRNAs may participate in honey bee memory by regulating learning [27]. The mushroom bodies of honey bee brains have significant levels of miR-276 expression [30]. The miR-2796 is highly expressed in honey bee brains, and binds to the phospholipase C-coding region. Phospholipase C is involved in the development and differentiation of mammalian neurons [51]. Hence, miR-2796 is speculated to be related to the behavioral plasticity of honey bees [52]. Behura and Whitfield discovered that miR-124, and miR-276 were up-regulated in nurse bees, whereas miR-210 was up-regulated in foragers. The different expression patterns of miRNAs between nurses and foragers indicate that miRNAs may play an important role in age-related behavioral changes in honey bees [53]. These three miRNAs were highly expressed in the samples with strong capabilities for a learner compared to a weak learner, indicating that miRNAs may play a crucial role in honey bee olfactory performance.

PER is a standard method, as a training mode, in an odor-associative learning experiment in honey bees [6]. The research suggests that honey bees less than 10 days old show a poorer performance in the PER training tests [34]. In our investigation, all the experimental bees were 12 days of age, did not become foragers, and were suitable for performing the PER experiments upon [54].

The data analysis revealed that some may be related to olfactory performance. The GO annotation and the KEGG enrichment analyses indicated that protein processing and signal transduction were critical to odor-learning behavior. The KEGG database annotation analysis of the target genes of DEmiRNA revealed that the two predicted genes (11.76%) were involved in neuroactive ligand-receptor interaction pathways. This pathway is divided into four subclasses according to ligand structure: class A (rhodopsin-like), class B (secretin-like), class C (metabotropic glutamate/pheromone), and channels/other receptors. The neuroactive ligand-receptor interaction pathway involves a set of receptors on the plasma membrane. It participates in the regulation of neuronal plasticity, learning, and memory [55]. The target genes that were annotated to the neuroactive ligand-receptor interaction pathway were N-methyl-D-aspartic acid receptor 2 (NR2, ID: LOC412818) and γ-aminobutyric acid (GABA) receptor subunit β (ID: LOC406124). Respectively, these were the predicted target genes of miR-275-3p and miR-2796-3p, they were related to transcription variants and GABA receptors. The N-methyl-D-aspartate (NMDA) receptor requires at least two subunits, NMDA receptor 1 and NR2, to function normally. These subunits are found in the NMDA receptors of mammals and insects and are transmitted by excitatory synapses in the brain and spinal cord [56]. NR2 recognizes glutamate and its other analogs. It regulates activated receptors and is inserted into the presynaptic and subsynaptic membranes. The NMDA receptors mediate olfactory learning and memory in *Drosophila melanogaster* [57]. The NMDA receptors are highly prominent structural sites in mushroom bodies that participate in nerve regulation [58,59]. The mushroom body is a crucial component that regulates learning and memory in honey bees [60]. Therefore, NR2 participates in the olfactory learning of honey bees. The GABA receptor subunit β is the receptor subunit of GABA and is abundant in the CNS of animals [61,62,63]. In honey bees, GABA is mainly released in the neurons of the antennal lobe and mushroom bodies and is crucial for olfactory information processing [64]. In a study of the olfactory loop, Hassani et al. described GABA and proved that GABA participates in olfactory learning and memory formation [63]. Olfactory learning assays showed that honey bees were more likely to learn a scent when it signaled a sucrose reward containing GABA and that GABA significantly enhanced the collection performance [65]. The miR-2796 is abundantly expressed in honey bee brains, and it is considered to be a key regulatory factor in neuron differentiation and development [51]. Some studies have shown that miR-275 and miR-2796 is up-regulated in the brains of nurses and foragers, respectively [52]. Among all of the miRNAs that were detected in the brains of honey bees, miR-2796 showed the highest expression in the weak group but not in the strong group. However, the miR-190-3p showed the lowest expression in the weak group. This expression pattern is thought to be related to the regulation of the nervous system development in honey bees.

## 5. Conclusions

DEmiRNAs and their target genes in the brains of *A. mellifera* with strong and weak olfactory performance were analyzed. The sequencing results revealed that 14 DEmiRNAs, including seven up-regulated and seven down-regulated miRNAs, were significantly different between the strong and weak learning performance groups. The expression of these miRNAs were validated using RT-qPCR. Among the 14 miRNAs, 9 miRNAs showed significant differences. The qPCR verification showed that four miRNAs were significantly differentially expressed and may regulate the olfactory performance of the honey bees. Meanwhile, 57 target genes were predicted based on the DEmiRNAs. The GO and KEGG pathways from the target genes of the identified miRNAs are likely to regulate the olfactory performance of the honey bees through the neuroactive ligand-receptor interaction pathway. Our results demonstrated that the identified miRNAs may regulate the olfactory performance of honey bees and provide the potential roles of miRNAs in the honey bee brains, which is helpful in obtaining a more comprehensive knowledge of the relationship between brain activity and bee behavior.

## Figures and Tables

**Table 1:** Quality control of data transcriptome.

Samples	Raw Reads	Clean Reads (%)	Q20	Q30
S1	10944423	10582740 (96.70%)	97.47%	92.37%
S2	10485737	10250570 (97.76%)	97.28%	91.85%
S3	10421125	10207059 (97.95%)	97.47%	91.70%
W1	10715404	10466355 (97.68%)	96.92%	91.02%
W2	10781488	10427751 (96.72%)	97.12%	91.29%
W3	10715404	10466355 (97.68%)	97.00%	90.79%

**Table 2:** Significant DEmiRNAs in the strong and weak olfactory performance with the number of potential target genes.

DEmiRNAs ID	Expression in S	Expression in W	log2 FC	PADJ	No. of Target Genes	Regulation
miR-184-3p	166,586.70	138,351.60	0.68	0.0014	4	Up
miR-2796-3p	113,690	91,192.20	0.72	0.0029	5	Up
miR-210-3p	2515.80	2436.06	0.47	0.011	16	Up
miR-87-3p	64,093.11	60,828.32	0.50	0.021	2	Up
miR-124-3p	656.06	625.88	0.47	0.043	10	Up
miR-275-3p	1153.24	1145.67	0.43	0.043	5	Up
miR-276-3p	342,060.70	290,130.80	0.66	0.00014	4	Up
miR-2944-3p	73.30	144.29	−0.50	0.017	5	Down
miR-9a-5p	2139.77	4322.34	−0.55	0.017	2	Down
miR-6062-3p	5.14	17.06	−0.91	0.018	1	Down
miR-965-3p	15.60	38.94	−0.73	0.018	2	Down
miR-971-3p	84.34	184.89	−0.60	0.045	1	Down
miR-190-5p	1476.59	2226.80	−0.56	0.0018	0	Down
miR-12-5p	469.26	672.82	−0.49	0.0176	0	Down

Abbreviation: DEmiRNAs; differentially expressed microRNAs; S: refers to the honeybees with strong olfactory performance; W: refers to the honeybees with weak olfactory performance; FC: fold change and PADJ: *p*-value after adjustment.

## Data Availability

The original sequence data were uploaded to the NCBI SRA database and the BioProject No. PRJNA724895.

## Supplementary Materials

The following supporting information can be downloaded at: https://www.mdpi.com/article/10.3390/genes14051000/s1, Figure S1: Honey bee demonstrating PER after touching 30% sucrose solution with its antenna; Figure S2: Scatter plot for KEGG enrichment results; Table S1: The most enriched pathway terms; Table S2: Primer sequences used for RT-qPCR; Table S3: RT-qPCR verification of DEmiRNAs statistics result.

## References

[1] Giurfa M. (2007). Behavioral and Neural Analysis of Associative Learning in the Honeybee: A Taste from the Magic Well. J. Comp. Physiol. A.

[2] Menzel R., Müller U. (1996). Learning and Memory in Honeybees: From Behavior to Neural Substrates. Annu. Rev. Neurosci..

[3] Chen Y.M., Fu Y., He J., Wang J.H. (2014). Effects of Cold Narcosis on Memory Acquisition, Consolidation and Retrieval in Honeybees (*Apis mellifera*). Zool. Res..

[4] Lichtenstein L., Brockmann A., Spaethe J. (2019). Learning of Monochromatic Stimuli in *Apis cerana* and *Apis mellifera* by Means of PER Conditioning. J. Insect Physiol..

[5] Stach S., Benard J., Giurfa M. (2004). Local-Feature Assembling in Visual Pattern Recognition and Generalization in Honeybees. Nature.

[6] Matsumoto Y., Menzel R., Sandoz J.C., Giurfa M. (2012). Revisiting Olfactory Classical Conditioning of the Proboscis Extension Response in Honey Bees: A Step toward Standardized Procedures. J. Neurosci. Methods..

[7] Giger A.D., Srinivasan M.V. (1995). Pattern Recognition in Honeybees: Eidetic Imagery and Orientation Discrimination. J. Comp. Physiol. A.

[8] Giurfa M., Sandoz J.C. (2012). Invertebrate Learning and Memory: Fifty Years of Olfactory Conditioning of the Proboscis Extension Response in Honeybees. Learn. Mem..

[9] Felsenberg J., Gehring K.B., Antemann V., Eisenhardt D. (2011). Behavioural Pharmacology in Classical Conditioning of the Proboscis Extension Response in Honeybees (*Apis mellifera*). J. Vis. Exp..

[10] Frost E.H., Shutler D., Hillier N.K. (2012). The Proboscis Extension Reflex to Evaluate Learning and Memory in Honeybees (*Apis mellifera*): Some Caveats. Naturwissenschaften.

[11] Faber T., Joerges J., Menzel R. (1999). Associative Learning Modifies Neural Representations of Odors in the Insect Brain. Nat. Neurosci..

[12] Edbauer D., Neilson J.R., Foster K.A., Wang C., Seeburg D.P., Batterton M.N., Tada T., Dolan B.M., Sharp P.A. (2010). Regulation of Synaptic Structure and Function by FMRP-associated MicroRNAs miR-125b and miR-132. Neuron.

[13] Wang Y.L., Yang M.L., Jiang F., Zhang J.Z., Kang L. (2013). MicroRNA-Dependent Development Revealed by RNA Interference-Mediated Gene Silencing of LmDicer1 in the Migratory Locust. Insect Sci..

[14] Picao-Osorio J., Johnston J., Landgraf M., Berni J., Claudio R. (2015). Europe PMC Funders Group MicroRNA-Encoded Behaviour in Drosophila. Science.

[15] Visvanathan J., Lee S., Lee B., Lee J.W., Lee S.K. (2007). The MicroRNA MiR-124 Antagonizes the Anti-Neural REST/SCP1 Pathway during Embryonic CNS Development. Genes Dev..

[16] Cristino A.S., Barchuk A.R., Freitas F.C.P., Narayanan R.K., Biergans S.D., Zhao Z., Simoes Z.L.P., Reinhard J., Claudianos C. (2014). Neuroligin-Associated MicroRNA-932 Targets Actin and Regulates Memory in the Honeybee. Nat. Commun..

[17] Michely J., Kraft S., Müller U. (2017). MiR-12 and MiR-124 Contribute to Defined Early Phases of Long-Lasting and Transient Memory. Sci. Rep..

[18] Alvarez-Garcia I., Miska E.A. (2005). MicroRNA Functions in Animal Development and Human Disease. Development.

[19] Liu F., Peng W., Li Z., Li W., Li L., Pan J., Zhang S., Miao Y., Chen S., Su S. (2012). Next-Generation Small RNA Sequencing for MicroRNAs Profiling in *Apis mellifera*: Comparison between Nurses and Foragers. Insect Mol. Biol..

[20] Stark A., Brennecke J., Bushati N., Russell R.B., Cohen S.M. (2005). Animal MicroRNAs Confer Robustness to Gene Expression and Have a Significant Impact on 3′UTR Evolution. Cell.

[21] Busto G.U., Guven-Ozkan T., Fulga T.A., Van Vactor D., Davis R.L. (2015). Micrornas That Promote or Inhibit Memory Formation in Drosophila Melanogaster. Genetics.

[22] Li W., Cressy M., Qin H., Fulga T., van Vactor D., Dubnau J. (2013). MicroRNA-276a Functions in Ellipsoid Body and Mushroom Body Neurons for Naive and Conditioned Olfactory Avoidance in Drosophila. J. Neurosci..

[23] Søvik E., Bloch G., Ben-Shahar Y. (2015). Function and Evolution of MicroRNAs in Eusocial Hymenoptera. Front. Genet..

[24] Lucas K., Raikhel A.S. (2013). Insect MicroRNAs: Biogenesis, Expression Profiling and Biological Functions. Insect Biochem. Mol. Biol..

[25] Yang M., Wei Y., Jiang F., Wang Y., Guo X., He J., Kang L. (2014). MicroRNA-133 Inhibits Behavioral Aggregation by Controlling Dopamine Synthesis in Locusts. PLoS Genet..

[26] Nunes F.M.F., Ihle K.E., Mutti N.S., Simões Z.L.P., Amdam G.V. (2013). The Gene Vitellogenin Affects MicroRNA Regulation in Honey Bee (*Apis mellifera*) Fat Body and Brain. J. Exp. Biol..

[27] Qin Q.H., Wang Z.L., Tian L.Q., Gan H.Y., Zhang S.W., Zeng Z.J. (2014). The Integrative Analysis of MicroRNA and mRNA Expression in *Apis mellifera* Following Maze-Based Visual Pattern Learning. Insect Sci..

[28] Shi T., Zhu Y., Liu P., Ye L., Jiang X., Cao H., Yu L. (2021). Age and Behavior-Dependent Differential MiRNAs Expression in the Hypopharyngeal Glands of Honeybees (*Apis mellifera* L.). Insects.

[29] Wang Z.L., Wang H., Qin Q.H., Zeng Z.J. (2013). Gene Expression Analysis Following Olfactory Learning in Apis mellifera. Mol. Biol. Rep..

[30] Hori S., Kaneko K., Saito T.H., Takeuchi H., Kubo T. (2011). Expression of Two MicroRNAs, Ame-Mir-276 and -1000, in the Adult Honeybee (*Apis mellifera*) Brain. Apidologie.

[31] Guo Y., Wang Z., Li Y., Wei G., Yuan J., Sun Y., Wang H., Qin Q., Zeng Z., Zhang S. (2016). Lateralization of Gene Expression in the Honeybee Brain during Olfactory Learning. Sci. Rep..

[32] Tsvetkov N., Cook C.N., Zayed A. (2019). Effects of Group Size on Learning and Memory in the Honey Bee Apis mellifera. J. Exp. Biol..

[33] Raza M.F., Anwar M., Husain A., Rizwan M., Li Z., Nie H., Hlaváč P., Ali M.A., Rady A., Su S. (2022). Differential Gene Expression Analysis Following Olfactory Learning in Honeybee (*Apis mellifera* L.). PLoS ONE.

[34] Li Z., Yu T., Chen Y., Heerman M., He J., Huang J., Nie H., Su S. (2019). Brain Transcriptome of Honey Bees (*Apis mellifera*) Exhibiting Impaired Olfactory Learning Induced by a Sublethal Dose of Imidacloprid. Pestic. Biochem. Physiol..

[35] Bitterman M.E., Menzel R., Fietz A., Schäfer S. (1983). Classical Conditioning of Proboscis Extension in Honeybees (*Apis mellifera*). J. Comp. Psychol..

[36] Kanazawa M., Endo M., Yamaguchi K., Hamaguchi T., Whitehead W.E., Itoh M., Fukudo S. (2005). Classical Conditioned Response of Rectosigmoid Motility and Regional Cerebral Activity in Humans. Neurogastroenterol. Motil..

[37] Li Z.G., Li M., Huang J.N., Ma C.S., Xiao L.C., Huang Q., Zhao Y.Z., Nie H.Y., Su S.K. (2017). Effects of Sublethal Concentrations of Chlorpyrifos on Olfactory Learning and Memory Performances in Two Bee Species, Apis mellifera and Apis cerana. Sociobiology.

[38] Li Z., Qiu Y., Li J., Wan K., Nie H., Su S. (2022). Chronic Cadmium Exposure Induces Impaired Olfactory Learning and Altered Brain Gene Expression in Honey Bees (*Apis mellifera*). Insects.

[39] Scheiner R., Abramson C.I., Brodschneider R., Crailsheim K., Farina W.M., Fuchs S., Grünewald B., Hahshold S., Karrer M., Koeniger G. (2013). Standard Methods for Behavioural Studies of Apis mellifera. J. Apic. Res..

[40] Baracchi D., Devaud J.M., D’Ettorre P., Giurfa M. (2017). Pheromones Modulate Reward Responsiveness and Non-Associative Learning in Honey Bees. Sci. Rep..

[41] Huang J., Zhang Z., Feng W., Zhao Y., Aldanondo A., de Brito Sanchez M.G., Paoli M., Rolland A., Li Z., Nie H. (2022). Food Wanting is Mediated by Transient Activation of Dopaminergic Signaling in the Honey Bee Brain. Science.

[42] Ghosh S., Chan C.K.K. (2016). Analysis of RNA-Seq Data Using TopHat and Cufflinks. Methods Mol. Biol..

[43] Langmead B., Trapnell C., Pop M., Salzberg S.L. (2009). Ultrafast and Memory-Efficient Alignment of Short DNA Sequences to the Human Genome. Genome Biol..

[44] Varet H., Brillet-Guéguen L., Coppée J.Y., Dillies M.A. (2016). SARTools: A DESeq2- and EdgeR-Based R Pipeline for Comprehensive Differential Analysis of RNA-Seq Data. PLoS ONE.

[45] Enright A.J., John B., Gaul U., Tuschl T., Sander C., Marks D.S. (2003). MicroRNA Targets in Drosophila. Genome Biol..

[46] Krüger J., Rehmsmeier M. (2006). RNAhybrid: MicroRNA Target Prediction Easy, Fast and Flexible. Nucleic Acids Res..

[47] Wu J., Mao X., Cai T., Luo J., Wei L. (2006). KOBAS Server: A Web-Based Platform for Automated Annotation and Pathway Identification. Nucleic Acids Res..

[48] Kondo T., Oka T., Sato H., Shinnou Y., Washio K. (2009). Accumulation of Aberrant CpG Hypermethylation by Helicobacter Pylori Infection Promotes Development. Int. J. Oncol..

[49] Finke V., Scheiner R., Giurfa M., Avarguès-Weber A. (2023). Individual Consistency in the Learning Abilities of Honey Bees: Cognitive Specialization within Sensory and Reinforcement Modalities. Anim. Cogn..

[50] Liu F., Shi T., Yin W., Su X., Qi L., Huang Z.Y., Zhang S., Yu L. (2017). The MicroRNA Ame-MiR-279a Regulates Sucrose Responsiveness of Forager Honey Bees (*Apis mellifera*). Insect Biochem. Mol. Biol..

[51] Wing M.R., Bourdon D.M., Harden T.K. (2003). PLC-epsilon: A shared effector protein in Ras-, Rho-, and G alpha beta gamma- mediated signaling. Mol. Interv..

[52] Greenberg J.K., Xia J., Zhou X., Thatcher S.R., Gu X., Ament S.A., Newman T.C., Green P.J., Zhang W., Robinson G.E. (2012). Behavioral Plasticity in Honey Bees Is Associated with Differences in Brain MicroRNA Transcriptome. Genes Brain Behav..

[53] Behura S.K., Whitfield C.W. (2010). Correlated Expression Patterns of MicroRNA Genes with Age-Dependent Behavioural Changes in Honeybee. Insect Mol. Biol..

[54] Raza M.F., Wang T., Li Z., Nie H., Giurfa M., Husain A., Hlaváč P., Kodrik M., Ali M.A., Rady A. (2022). Biogenic Amines Mediate Learning Success in Appetitive Odor Conditioning in Honeybees. J. King Saud Univ. Sci..

[55] Su S.Y., Hsieh C.L., Wu S.L., Cheng W.Y., Li C.C., Lo H.Y., Ho T.Y., Hsiang C.Y. (2009). Transcriptomic Analysis of EGb 761-Regulated Neuroactive Receptor Pathway in Vivo. J. Ethnopharmacol..

[56] Mcbain C.J., Mayer M.L. (1994). N-Methyl-D-Aspartic Acid Receptor Structure and Function. Physiol. Rev..

[57] Xia S., Miyashita T., Fu T.F., Lin W.Y., Wu C.L., Pyzocha L., Lin I.R., Saitoe M., Tully T., Chiang A.S. (2005). NMDA Receptors Mediate Olfactory Learning and Memory in Drosophila. Curr. Biol..

[58] Frambach I., Rössler W., Winkler M., Schürmann F.W. (2004). F-Actin at Identified Synapses in the Mushroom Body Neuropil of the Insect Brain. J. Comp. Neurol..

[59] Zachepilo T.G., Il’Inykh Y.F., Lopatina N.G., Molotkov D.A., Popov A.V., Savvateeva-Popova E.V., Vaido A.I., Chesnokova E.G. (2008). Comparative Analysis of the Locations of the NR1 and NR2 NMDA Receptor Subunits in Honeybee (*Apis mellifera*) and Fruit Fly (*Drosophila Melanogaster*, Canton-S Wild-Type) Cerebral Ganglia. Neurosci. Behav. Physiol..

[60] Sandoz J.C. (2011). Behavioral and Neurophysiological Study of Olfactory Perception and Learning in Honeybees. Front. Syst. Neurosci..

[61] Huang Q.T., Sheng C.W., Jiang J., Jia Z.Q., Han Z.J., Zhao C.Q., Liu G.Y. (2019). Functional Integrity of Honeybee (*Apis mellifera* L.) Resistant to Dieldrin γ-Aminobutyric Acid Receptor Channels Conjugated with Three Fluorescent Proteins. Insect Mol. Biol..

[62] Dupuis J.P., Bazelot M., Barbara G.S., Paute S., Gauthier M., Raymond-Delpech V. (2010). Homomeric RDL and Heteromeric RDL/LCCH3 GABA Receptors in the Honeybee Antennal Lobes: Two Candidates for Inhibitory Transmission in Olfactory Processing. J. Neurophysiol..

[63] El Hassani A.K., Giurfa M., Gauthier M., Armengaud C. (2008). Inhibitory Neurotransmission and Olfactory Memory in Honeybees. Neurobiol. Learn. Mem..

[64] Boitard C., Devaud J.M., Isabel G., Giurfa M. (2015). GABAergic Feedback Signaling into the Calyces of the Mushroom Bodies Enables Olfactory Reversal Learning in Honey Bees. Front. Behav. Neurosci..

[65] Carlesso D., Smargiassi S., Pasquini E., Bertelli G., Baracchi D. (2021). Nectar Non-Protein Amino Acids (NPAAs) Do Not Change Nectar Palatability but Enhance Learning and Memory in Honey Bees. Sci. Rep..

