# Supplementary material for: Differential Brain Expression Patterns of microRNAs Related to Olfactory Performance in Honey Bees (Apis mellifera)
Source: Genes (Basel). 2023 Apr 28;14(5):1000. doi: 10.3390/genes14051000 (PMC10217982; doi:10.3390/genes14051000)
Supplement: Supplementary file 1 [file genes-14-01000-s001.zip › Figure S1. Honey bee demonstrating PER after touching 30% sucrose solution with its antenna.pdf]

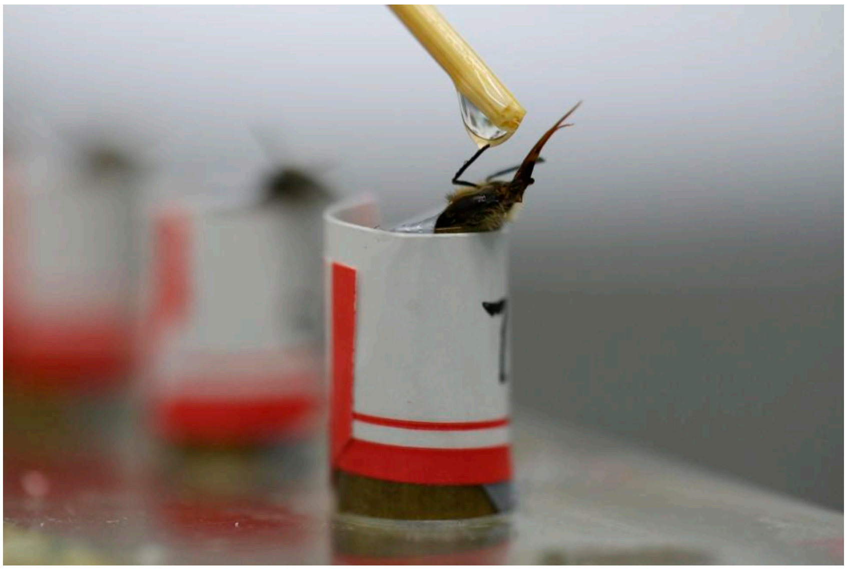

Figure S1. Honey bee demonstrating PER after touching 30% sucrose solution with its antenna.
Note: PER of a harnessed *A. mellifera* to antennal stimulation with 30% sucrose solution.
