# Supplementary material for: Differential Brain Expression Patterns of microRNAs Related to Olfactory Performance in Honey Bees (Apis mellifera)
Source: Genes (Basel). 2023 Apr 28;14(5):1000. doi: 10.3390/genes14051000 (PMC10217982; doi:10.3390/genes14051000)
Supplement: Supplementary file 1 [file genes-14-01000-s001.zip › Figure S2. Scatter plot for KEGG enrichment results.pdf]

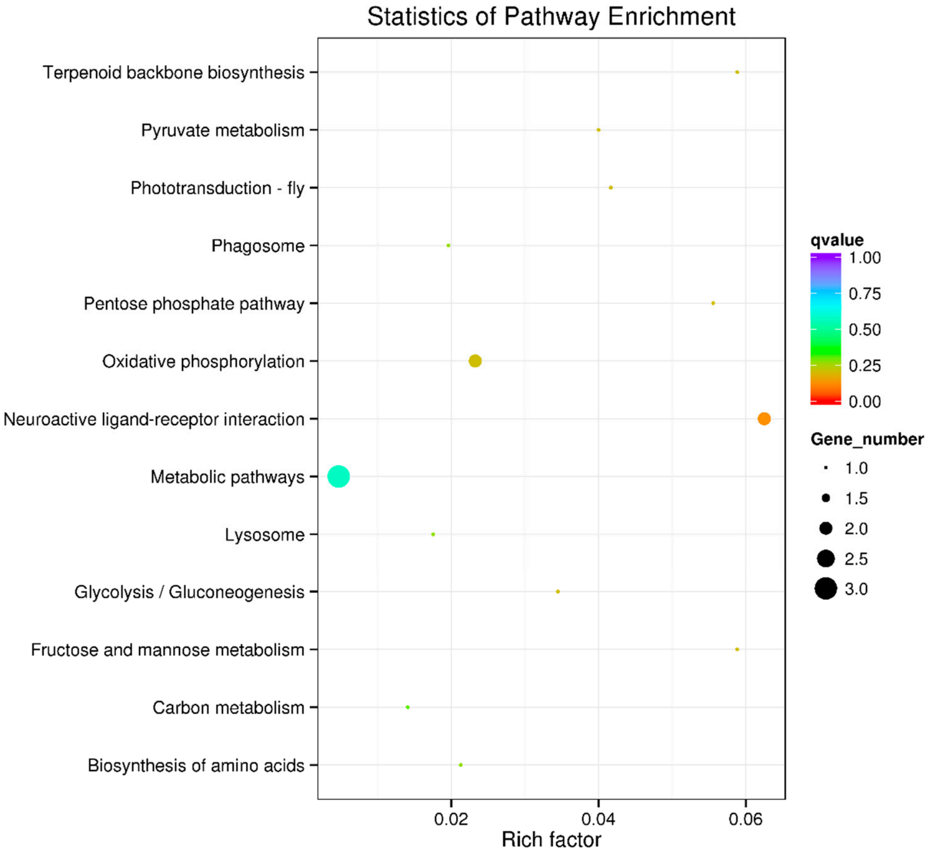

Figure S2. Scatter plot for KEGG enrichment results. The Rich factor is the ratio of DEG numbers annotated in this pathway term to all gene numbers annotated in this pathway term. The greater the Rich factor, the greater the degree of pathway enrichment. A q-value is the corrected p value ranging from 0 to 1, and a lower value indicates greater pathway enrichment.
