# Supplementary material for: Differential Brain Expression Patterns of microRNAs Related to Olfactory Performance in Honey Bees (Apis mellifera)
Source: Genes (Basel). 2023 Apr 28;14(5):1000. doi: 10.3390/genes14051000 (PMC10217982; doi:10.3390/genes14051000)
Supplement: Supplementary file 1 [file genes-14-01000-s001.zip › Table S1.The most enriched pathway terms.pdf]

Table S1. The most enriched pathway terms

Term	Sample number	Background number	*p*-value	Corrected *p*-value	Uni-Genes	KO	Entrez ID
Neuroactive ligand-receptor interaction	2	32	0.0098	0.13	412818 406124	ame:412818 ame:406124	412818 406124
Oxidative phosphorylation	2	86	0.058	0.2	413891 412810	ame:413891 ame:412810	413891 412810
Fructose and mannose metabolism	1	17	0.078	0.2	725455	ame:725455	725455
Terpenoid backbone biosynthesis	1	17	0.078	0.2	551463	ame:551463	551463
Pentose phosphate pathway	1	18	0.082	0.2	725455	ame:725455	725455
Phototransduction - fly	1	24	0.11	0.2	413224	ame:413224	413224
Pyruvate metabolism	1	25	0.11	0.2	724904	ame:724904	724904
Glycolysis / Gluconeogenesis	1	29	0.13	0.2	725455	ame:725455	725455
Biosynthesis of amino acids	1	47	0.21	0.27	725455	ame:725455	725455
Phagosome	1	51	0.21	0.27	412810	ame:412810	412810
Lysosome	1	57	0.23	0.27	412810	ame:412810	412810
Carbon metabolism	1	71	0.28	0.3	725455	ame:725455	725455
Metabolic pathways	3	637	0.57	0.57	725455 413891 412810	ame:725455 ame:413891 ame:412810	725455 413891 412810

Note: Statistic method hypergeometric test /Fisher's exact test with *p*-value < 0.05 was considered significantly enriched and false discovery rate (FDR) correction method Benjamini and Hochberg was to detect the most enrichments pathway terms.
