# Supplementary material for: Differential Brain Expression Patterns of microRNAs Related to Olfactory Performance in Honey Bees (Apis mellifera)
Source: Genes (Basel). 2023 Apr 28;14(5):1000. doi: 10.3390/genes14051000 (PMC10217982; doi:10.3390/genes14051000)
Supplement: Supplementary file 1 [file genes-14-01000-s001.zip › Table S2. Primer sequences used for RT-qPCR.pdf]

Table S2. List of Primers used in qPCR

Primer name	Primer sequence (5′ to 3′)	
ame-miR-87-3p-F	GCGCGCGGTGAGCAAAGTT	
ame-miR-87-3p-Loop	CTCAACTGGTGTCGTGGAGTCGGCAATTCAGTTGAGACACCTGA	
ame-miR-124-3p-F	GCGCGCGTAAGGCACGCGGTGA	
ame-miR-124-3p-Loop	CTCAACTGGTGTCGTGGAGTCGGCAATTCAGTTGAGCTTGGCAT	
ame-miR-275-3p-F	GCGCGCGTCAGGTACCTGAAGT	
ame-miR-275-3p-Loop	CTCAACTGGTGTCGTGGAGTCGGCAATTCAGTTGAGCGCGCGCT	
ame-miR-190-5p-F	GCGCGCGAGATATGTTTGATATTCT	
ame-miR-190-5p-Loop	CTCAACTGGTGTCGTGGAGTCGGCAATTCAGTTGAGAACAACCA	
ame-miR-2944-3p-F	GCGCGCGTATCACAGCAGTAGT	
ame-miR-2944-3p-Loop	CTCAACTGGTGTCGTGGAGTCGGCAATTCAGTTGAGACCAGGTA	
ame-miR-9a-5p-F	GCGCGCGTCTTTGGTTATCTAG	
ame-miR-9a-5p-Loop	CTCAACTGGTGTCGTGGAGTCGGCAATTCAGTTGAGTCATACAG	
ame-miR-12-5p-F	GCGCGCGTGAGTATTACATCAG	
ame-miR-12-5p-Loop	CTCAACTGGTGTCGTGGAGTCGGCAATTCAGTTGAGACCAGTAC	
ame-miR-6062-3p-F	GCGCGCGTACGATATTGGTAT	
ame-miR-6062-3p-Loop	CTCAACTGGTGTCGTGGAGTCGGCAATTCAGTTGAGTGTCCTAA	
ame-miR-965-3p-F	GCGCGCGTAAGCGTATAGCTT	
ame-miR-965-3p-Loop	CTCAACTGGTGTCGTGGAGTCGGCAATTCAGTTGAGAAGGGAAA	
me-miR-971-3p-F	GCGCGCGTTGGTGTTCTACCTT	
me-miR-971-3p-Loop	CTCAACTGGTGTCGTGGAGTCGGCAATTCAGTTGAGCTCACTGT	
ame-miR-184-3p-F	GCGCGCGTGGACGGAGAACTG	
ame-miR-184-3p-Loop	CTCAACTGGTGTCGTGGAGTCGGCAATTCAGTTGAGGCCCTTAT	
ame-miR-276-3p-F	GCGCGCGTAGGAACTTCATAC	
ame-miR-276-3p-Loop	CTCAACTGGTGTCGTGGAGTCGGCAATTCAGTTGAGAGAGCACG	
ame-miR-2796-3p-F	GCGCGCGGTAGGCCGGCGGAAA	
ame-miR-2796-3p-Loop	CTCAACTGGTGTCGTGGAGTCGGCAATTCAGTTGAGGCAAGTAG	
ame-miR-210-3p-F	GCGCGCGTTGTGCGTGTGAC	
ame-miR-210-3p-Loop	CTCAACTGGTGTCGTGGAGTCGGCAATTCAGTTGAGTAGCCGCT	
mQ Primer R	GTCGTATCCAGTGCAGGGTCCGAGGTATTCGCACTGGATACGAC	
F-RpL32 (Reference gene)	CGATCTCGCCGCAGTAAAC	
F-U6 (Reference gene)	GTTAGGCTTTGACGATTTCG	[58]
R-U6	GGCATTTCTCCACCAGGTA	

Note: All these primers were designed in this study for the reference genes; the primers were used from published papers.
