# Supplementary material for: Differential Brain Expression Patterns of microRNAs Related to Olfactory Performance in Honey Bees (Apis mellifera)
Source: Genes (Basel). 2023 Apr 28;14(5):1000. doi: 10.3390/genes14051000 (PMC10217982; doi:10.3390/genes14051000)
Supplement: Supplementary file 1 [file genes-14-01000-s001.zip › Table S3. RT-qPCR verification of DEmiRNAs statistics result.pdf]

Table S3. RT-qPCR verification of DEmiRNAs statistics result

**miRNA name**	***t*-value**	***p*-value**
ame-miR-87-3P	3.81	0.0034
ame-miR-276-3P	2.43	0.036
ame-miR-12-3P	1.84	0.095
ame-miR-124-5p	2.15	0.05
ame-miR-2796-3p	-1.23	0.25
ame-miR-2944-3p	2.17	0.05
ame-miR-184-3p	2.37	0.041
ame-miR-275-3p	1.56	0.14
ame-miR-965-3p	2.17	0.05
ame-miR-971-3p	2.42	0.036
ame-miR-9a-5p	2.26	0.048
ame-miR-210-3p	1.12	0.29
ame-miR-190-5p	2.24	0.049

Note: Comparing the difference between weak and strong olfactory performance. Independent sample *t*-tests with *p*-values ≤ 0.05 was considered significantly different.
